# Workplace mortality risk and social determinants among migrant workers: a systematic review and meta-analysis

**DOI:** 10.1016/S2468-2667(24)00226-3

**Published:** 2019-06-17

**Authors:** Karen Lau, Robert Aldridge, Marie Norredam, George Frederick Mkoma, Mathura Kugan, Rosita Chia-Yin Lin, Ligia Kiss, Cathy Zimmerman, Sally Hargreaves

**Affiliations:** a The Migrant Health Research Group and the Consortium for Migrant Worker Health, Institute for Infection and Immunity, City St George's, University of London, London, UK; b Department of Global Health and Development, Faculty of Public Health and Policy, London School of Hygiene & Tropical Medicine, London, UK; c Institute for Health Metrics and Evaluation, University of Washington, Seattle, WA, USA; d Danish Research Centre for Migration, Ethnicity and Health, Section of Health Services Research, Department of Public Health, University of Copenhagen, Copenhagen, Denmark; e Section of Immigrant Medicine, Department of Infectious Diseases, Hvidovre University Hospital, Hvidovre, Denmark; f Department of Epidemiology Research, Statens Serum Institut, Copenhagen, Denmark

## Abstract

**Background:**

Migrant workers, a population of 170 million, often work in dangerous or unhealthy working environments and are likely to suffer workplace injuries and labour abuses. However, the risk of mortality in migrant workers compared with local workers is unknown. We aim to synthesise global evidence on migrant worker mortality risk and identify social determinants to inform health and safety protections for migrant workers.

**Methods:**

We conducted a systematic review and meta-analysis of peer-reviewed literature to examine mortality outcomes among migrant workers and associated risk factors. We searched MEDLINE, Embase, PsycINFO, and Ovid Global Health for studies published between Jan 1, 2000, and Jan 17, 2023, reporting quantitative primary research in English. A broad definition of migrant worker was used, including any worker who is foreign-born (ie, international first-generation migrant workers), either in paid employment or self-employment. Internal migrants, second-generation migrants, and foreign health-care workers were excluded. The primary outcome was any reported mortality, including all-cause mortality, cause-specific mortality, suicide, homicide, and fatal occupational injury. We used meta-analysis to compare outcomes between migrant worker and local worker populations, and a random-effects model to calculate pooled estimates. We used narrative synthesis to develop a data-driven conceptual framework capturing the intersectional social determinants of mortality in migrant workers. The study protocol is registered on PROSPERO, CRD42023372893.

**Findings:**

Of 11 495 identified records, 44 were included in the systematic review, of which 11 studies were pooled in meta-analyses. Data were from 16 countries, most of which were high-income countries, and included 44 338 migrant worker deaths, including migrants from the agriculture, construction, mining, and service industries. Compared with local workers, migrant workers had a higher risk of fatal occupational injury (pooled relative risk 1·71, 95% CI 1·22–2·38; eight studies; *I*^2^=99·4%), and a lower risk of all-cause mortality (0·94, 0·88–0·99; three studies, *I*^2^=90·7%). Migrant workers were more likely to die from external causes of death (such as falls or assaults) than internal causes of death (such as respiratory or digestive diseases) compared with local workers, with migrant workers also more likely to die from work-related homicides, especially in the retail and sex industries, with some evidence of higher suicide rates among female migrant workers compared with female local workers. Influential social determinants for poor fatality outcomes include migration-related factors (such as lower language proficiency, undocumented status, and long duration of stay) and labour-related factors (such as precarious employment, labour migration policies, and economic deregulation policies).

**Interpretation:**

Migrant workers have a higher risk of workplace fatal injury despite being generally healthier than local workers, which could be explained by structural determinants such as precarious employment and inadequate safety protection. This health inequity must be urgently addressed through future interventions that account for migration-related and labour-related social determinants of health at the structural level, such as extending labour protection laws to migrant workers, and improving occupational health and safety and workplace conditions for this vital and growing workforce.

**Funding:**

UK Medical Research Council and National Institute for Health and Care Research.

## Introduction

The global migrant workforce, estimated at 170 million people, represents 5% of the labour force. The number is rising, with Europe having the largest share.^1^ The COVID-19 pandemic and the 2022 Qatar FIFA World Cup have exposed the disproportionate burden of disease and injury experienced by migrant workers.^2^, ^3^ Migrants are more likely to work in high-risk sectors, be exposed to work hazards, have weaker labour and social protection, and have lower access to health care, which increases their physical and mental health risks.^4^ A 2023 *Lancet* Series on work and health, as well as a 2023 report from WHO, have identified labour migration as a research priority.^5^, ^6^

**Research in context**

**Evidence before this study**

Migrant workers, a population of 170 million, sustain the global economy and maintain the functioning of essential services. Yet, many work in dangerous or unhealthy working environments and are likely to suffer workplace injuries, illness, and labour abuses. As the largest mobile population and a vital workforce, there has been little evidence on their risk of workplace fatalities. UN Sustainable Development Goal indicator 8.8.1 specifically calls for countries to report rates of fatal occupational injuries disaggregated by migrant status, yet only 10% of countries have reported any data. Estimates from the International Labour Organization on the global work-related burden of disease and injury suggest that 2·9 million deaths in 2019 were linked to the workplace. However, these estimates were not disaggregated by migration status. We searched MEDLINE, Embase, PsycINFO, and Ovid Global Health for systematic reviews in English published between Jan 1, 2000, and Jan 17, 2023, using broad search terms related to migrant workers and mortality. Our 2018 systematic review for the *UCL–*Lancet *Commission on Migration and Health*, comparing general populations of migrants versus local residents, found an overall all-cause mortality advantage in all migrant populations; however, the findings did not consider how work and work conditions might have affected this advantage. When examining work-related injuries, our 2019 systematic review found high rates of injuries among migrant workers, including falls from height, fractures, dislocations and ocular injuries, and another systematic review by Pega and colleagues (2021) found inconsistent evidence on the risk of fatal occupational injuries compared with non-migrant workers. Of note, findings are not disaggregated by age or gender. This study aims to synthesise global evidence on migrant worker mortality risk compared with local workers and identify social determinants to inform health and safety protections for migrant workers.

**Added value of this study**

This global study (involving 44 338 migrant worker deaths compiled from 16 countries) generates the strongest evidence to date that migrant workers have a higher risk of fatal occupational injury than local workers. Migrant workers are more likely to die from external causes of death (such as falls or assaults) than internal causes of death (such as respiratory diseases or digestive diseases) compared with local workers. We also show that migrant workers have a lower risk of all-cause mortality compared with local workers. Additionally, we propose a data-driven framework to explicate the intersectionality between migration-related and labour-related factors at the structural level, along a temporal dimension corresponding to the stages of migration now considered fundamental to migration and health research.

**Implications of all the available evidence**

This study is a timely response to recent international calls to prioritise migrant worker health research, including from WHO and a *Lancet* Series on work and health. The results highlight that migrants have higher risks of death at work, emphasising the need for strategies that address these health inequalities. The data-driven conceptual framework suggests intervention priorities to guide future interventions and policy measures to reduce injuries and deaths among migrant workers. Targeted preventive measures at both the individual level (such as language-sensitive safety training) and structural level (such as labour protection laws and compensation mechanisms for migrant workers) should be coordinated to promote a safe and healthy working environment for migrant workers. To meet our international UN obligations for decent work and health for all, there is an urgent need to improve global measurement of migrant workers’ health and to strengthen migrant-inclusive occupational health and safety measures.

Despite this growing attention, the global burden of mortality in migrant workers remains unknown. It is unclear whether migrant workers have a higher risk of mortality compared with local workers, and data are scarce and inconsistent. Our previous systematic review, published as part of the *UCL–*Lancet *Commission on Migration and Health*,^7^ found that migrants had an overall mortality advantage (ie, lower death rates) compared with host populations, in line with the healthy migrant effect, yet no conclusions specific to migrant workers could be drawn.^8^ Another systematic review found high rates of injuries among migrant workers globally, including falls from height, fractures, dislocations, and ocular injuries, but did not focus on mortality.^9^ A 2021 systematic review on migrant workers’ health found inconsistent evidence on fatal occupational injuries, with few age-specific or sex-specific findings.^10^ Thus, the literature seems to suggest a somewhat paradoxical observation in which people who migrate tend to demonstrate an overall health advantage compared with local populations, but at the same time this group might be especially vulnerable to occupational injury and disease. Building on these previous findings, our systematic review focuses specifically on mortality outcomes among migrant workers, expanding the types of mortality outcome studied to those beyond fatal occupational injury. Work has been inadequately considered as a fundamental social determinant of health inequalities, and the health impacts of labour exploitation among the low-wage workforce—specifically including migrants—has been widely neglected in health research so far.^5^

WHO and the International Labour Organization (ILO) estimated that 1·88 million deaths globally in 2016 were attributable to occupational risk factors,^11^ which increased to 2·93 million work-related deaths in 2019, estimated by the ILO.^12^ However, estimates were not disaggregated by migration status. Sustainable Development Goal (SDG) indicator 8.8.1 specifically calls for countries to report rates of fatal and non-fatal occupational injuries by migrant status,^13^ yet only 10% of countries reported any such data since 2000, and current data suffer from poor quality and comparability.^14^ We did a systematic review and meta-analysis of global evidence to evaluate whether migrant workers have a higher risk of mortality than local workers and to identify the social determinants associated with mortality.

## Methods

### Search strategy and selection criteria

This systematic review and meta-analysis examined global peer-reviewed literature on migrant worker mortality risk. We searched four databases (MEDLINE, Embase, PsycINFO, and Ovid Global Health). The search strategy is available in the appendix (pp 1–2). We included English-language studies published from Jan 1, 2000, to Jan 17, 2023. All studies reporting quantitative primary research were included. Commentaries, case studies, qualitative studies, reviews, and grey literature were excluded.

A migrant worker in this study was defined as “a person who is to be engaged, is engaged or has been engaged in a remunerated activity in a State of which he or she is not a national”, in accordance with the UN Convention on the Protection of the Rights of all Migrant Workers and Members of their Families.^15^ To ensure that no key literature was excluded, we adopted a broader inclusion criterion by including any worker who is foreign-born (ie, international first-generation migrant workers), either in paid employment or self-employment. This definition included refugees, asylum seekers, regular migrants, irregular migrants, and undocumented migrants who worked. Internally displaced people, internal migrants, family members, non-working migrants, and second-generation migrants were excluded. Foreign health-care workers were also excluded because they are highly skilled and have specialised knowledge in health care, and are therefore likely to have a different mortality risk profile than other migrant workers. Comparison groups included any non-migrant working population. Subgroup analysis compared subpopulations within the migrant worker population. We defined high-income, middle-income, and low-income countries according to the World Bank criteria.^16^

Two reviewers (KL, MK, or GFM) independently screened records at the title, abstract, and full-text screening stages. Conflicts were resolved by consensus. Rayyan was used for recording decisions, including reasons for exclusion during full-text screening. The study protocol was developed based on PRISMA-P guidelines and is registered on PROSPERO, CRD42023372893. No changes have been made from the protocol in the conduct of this systematic review.

### Data analysis

The primary outcome was any reported mortality, including all-cause mortality, cause-specific mortality, suicide, homicide, and fatal occupational injury. We excluded maternal or perinatal outcomes and existing conditions, which are less related to the work environment and more related to access to care. COVID-19-related outcomes were excluded because there is a growing body of literature summarising these outcomes in migrant populations.^17^ Suicide attempts or intent that did not result in death were excluded. Exposures included risk factors at the individual level (sex or gender and legal status) and structural level (employment terms and policies). Studies that only reported on risk factors without reporting on quantitative mortality risks were excluded.

We developed Excel templates for data extraction, covering study characteristics, participants, comparison groups, and results. One reviewer extracted data from the included studies (KL), which was cross-checked by another reviewer (GFM). All quantitative data reporting mortality either as absolute or relative risks were extracted. Numerators, denominators, CIs, and SEs were extracted. Where missing, relative risks along with SEs were computed using mathematical conversion formulae from Cochrane. If a study reported more than one relative risk by migrant worker subgroup, we combined these into a single pooled relative risk, along with its SE, using meta-analysis with a random-effects model, such that each individual study only contributed one relative risk estimate for subsequent meta-analyses. Statistical heterogeneity between included studies was assessed using the *I*^2^ statistic. Studies in which local workers were used as the comparison group were combined using meta-analysis if studies were considered sufficiently homogeneous based on clinical heterogeneity (differences in study population) and methodological heterogeneity (differences in study design). A random-effects model was used to calculate pooled estimates along with 95% CIs using R (version 4.2.2) and presented as forest plots. Sensitivity analyses were conducted to assess the effect of using a fixed-effects model instead of a random-effects model. For the secondary outcome of risk factors, either quantitative or qualitative data were extracted. We conducted subgroup analyses using narrative synthesis to identify social determinants of mortality and developed a conceptual framework.

Two reviewers (KL and MK) independently assessed the risk of bias of included studies using the adapted version of the Newcastle–Ottawa Scale.^8^ Discrepancies were resolved by consensus. Studies were deemed low quality if they scored less than 50% (high risk of bias), medium quality if they scored between 50% and 75% (moderate risk of bias), and high quality if they scored above 75% (low risk of bias). We conducted a sensitivity analysis excluding low-quality studies in meta-analyses. We used funnel plots to assess risk of publication bias.

### Role of the funding source

The funders of the study had no role in study design, data collection, data analysis, data interpretation, or writing of the report.

## Results

**Figure 1:**
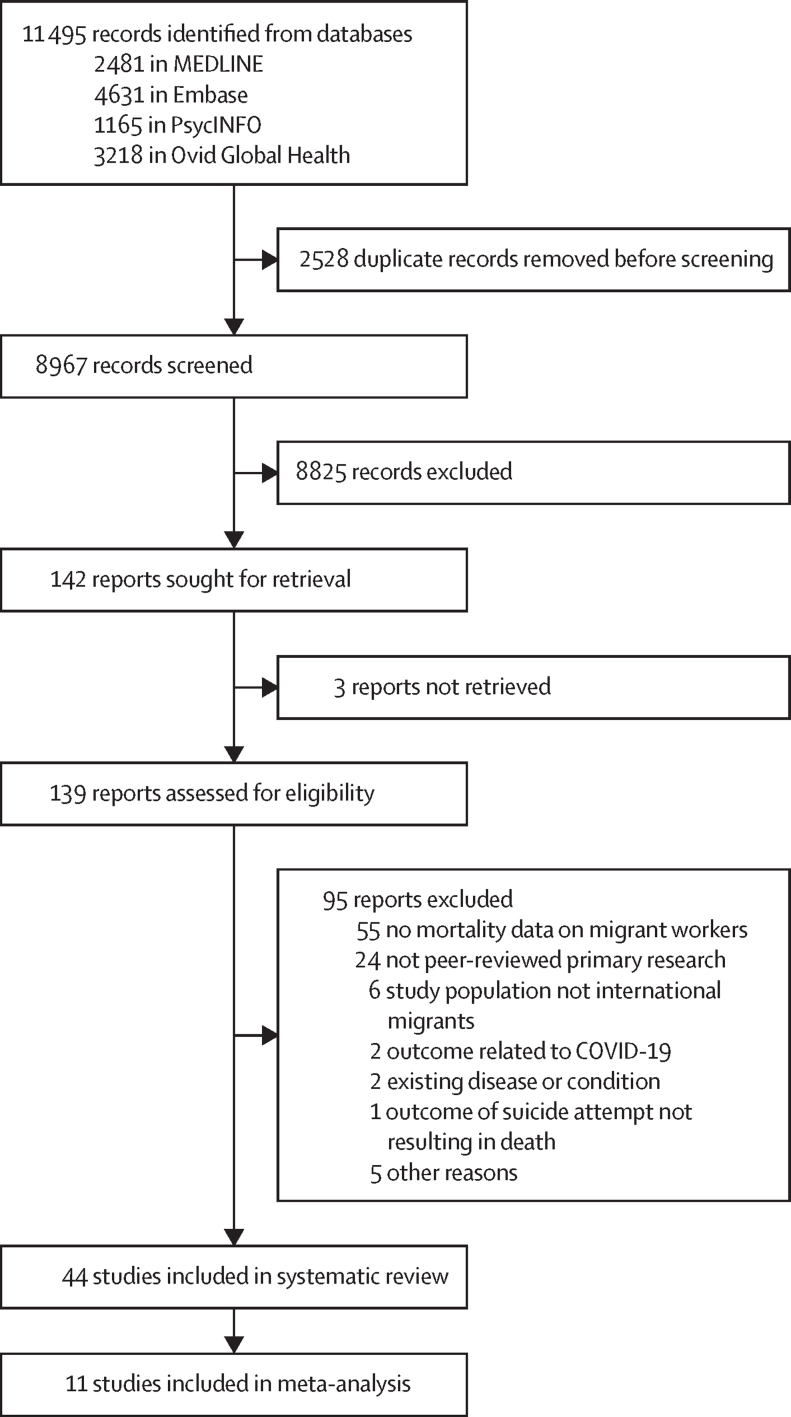
Study selection

Of 11 495 identified records, 8967 were screened, 139 were assessed for eligibility, and 44 studies were included,^18^, ^19^, ^20^, ^21^, ^22^, ^23^, ^24^, ^25^, ^26^, ^27^, ^28^, ^29^, ^30^, ^31^, ^32^, ^33^, ^34^, ^35^, ^36^, ^37^, ^38^, ^39^, ^40^, ^41^, ^42^, ^43^, ^44^, ^45^, ^46^, ^47^, ^48^, ^49^, ^50^, ^51^, ^52^, ^53^, ^54^, ^55^, ^56^, ^57^, ^58^, ^59^, ^60^, ^61^ with 11 included in the meta-analyses^18^, ^22^, ^24^, ^27^, ^34^, ^36^, ^38^, ^47^, ^48^, ^53^, ^58^ (figure 1). The total number of migrant worker deaths (where data were available) was 44 338, including in the agriculture, construction, mining, and service industries. Data were from 16 countries, with 42 studies (95%) from high-income countries (16 from the USA, four from Spain, four from Sweden, three from Australia, three from Canada, two from Singapore, two from South Korea, two from Qatar, and one each from Belgium, Germany, Japan, Norway, United Arab Emirates, and the UK), two (5%) from middle-income countries (one each from Sri Lanka and Türkiye), and none from low-income countries (table). All studies were observational studies, with 29 (66%) using a cohort-based design and 33 (75%) using a reference population of local workers. Mortality data were available on fatal occupational injuries, all-cause mortality, and other cause-specific mortality.

**Table:** Characteristics of included studies

	**Country**	**Years of study**	**Study design**	**Sample size of migrant workers**	**Sample size of local workers**	Male, n (%); female, n (%)*	**Study population**	**Outcome**	**Reference population**	**Migrant worker definition**	**Country or region of origin of migrant workers**	**Sector**	**Quality of study**†
Ahonen and Benavides (2006)^18^	Spain	2003	Retrospective observational	322 deaths; 747 537 at risk	1099 deaths; 12 948 163 at risk	1313 (92·4%); 108 (7·6%)	Insured workers registered for occupational injury with the Ministry of Labour and Social Issues	Fatal occupational injury	Spanish workers	Non-national workers	NA	NA	86%
Al-Thani et al (2015)^19^	Qatar	2010–13 (data on rates available for 2012 only)	Retrospective cohort	86 deaths; 1 257 981 at risk	No deaths; 82 601 at risk	1972 (97·9%); 43 (2·1%)	Patients with occupational injuries aged ≥18 years admitted to national tertiary trauma centre	Fatal occupational injury	Qatari workers	Non-national workers	India, Nepal, Philippines, and others	NA	75%
Arndt et al (2004)^20^	Germany	1986–2000	Prospective cohort	153 deaths; 5102 at risk	663 deaths; 14 725 at risk	818 (100%); 0	Male construction workers aged 25–64 years who underwent occupational health examinations	All-cause mortality; cause-specific mortality	German workers	Non-national workers	Former Yugoslavia, Italy, Turkey, and others	Construction	88%
Baraza and Cugueró-Escofet (2022)^21^	Spain	2013–18	Retrospective observational	NA	NA	115 774 (80·3%); 28 491 (19·7%)	Workers with reported occupational accidents in the agricultural sector	Fatal occupational accident	Spanish workers	Non-national workers	Morocco, Romania, Ecuador, and others	Agriculture	57%
Byler and Robinson (2018)^22^	USA	2003–10	Retrospective cohort	7096 deaths; population at risk NA	31 952 deaths; population at risk NA	36 084 (92·4%); 2964 (7·6%)	Workers aged ≥16 years	Fatal work injury (excludes fatal illness); in this survival analysis, life expectancy refers to the expectation of being able to work until retirement age	US-born workers	Foreign-born workers	Any	NA	88%
Carangan et al (2004)^23^	Singapore	1998–99	Retrospective cohort	3 deaths; 1936 at risk	No deaths; 1244 at risk	2909 (91·5%); 271 (8·5%)	Patients >15 years with work-related injury who presented to the emergency department	Death in hospital	Singaporean workers	Non-national or non-permanent resident workers	NA	NA	50%
Cha and Cho (2014)^24^	South Korea	2005–07	Retrospective cohort	255 deaths; 942 817 at risk	7352 deaths; 35 276 869 at risk	235 618 (83·3%); 47 347 (16·7%)	Workers compensated by the National Occupational Injury Compensation Insurance	Fatal occupational injury; fatal occupational disease	Korean workers	Non-national workers	China, Viet Nam, Mongolia, Thailand, Philippines, and others	All, manufacturing, construction, and others	75%
Chiu et al (2022)^25^	Singapore	2011–14	Retrospective observational	232 deaths; population at risk NA	540 deaths; population at risk NA	1222 (67·5%); 588 (32·5%)	Suicide cases	Suicide	Singaporean workers	Non-national or non-permanent resident workers	Any	Not specified	71%
Cooper et al (2001)^26^	USA	1984–96	Prospective cohort	15 deaths; 196 at risk	None	100 (51·0%); 96 (49·0%)	Mexican American migrant farmworkers in Texas who participated in earlier studies	All-cause mortality	None	Self-classified migrant farm workers	Mexico	Agriculture	63%
Cruz et al (2018)^27^	USA	2001–14	Retrospective cohort	87 deaths; population at risk NA	1361 deaths; population at risk NA	1342 (92·7%); 106 (7·3%)	Workers in Kentucky	Fatal occupational injury; external cause of mortality	US-born workers	Foreign-born workers	Any	NA	38%
Cunningham et al (2018)^28^	UK	1990–2016	Retrospective observational	13 deaths; population at risk NA	85 deaths; population at risk NA	2 (1·8%); 105 (95·5%)‡	Sex workers	Occupational homicide	UK-born workers	Foreign-born workers	NA	Sex work	43%
Dávila et al (2011)^29^	USA	1999–2000	Retrospective observational	NA	NA	NA (100%); 0	Male Hispanic workers aged 25–64 years	Occupational injury fatality	US-born Hispanic and non-Hispanic workers	Foreign-born Hispanic workers	NA	Not specified	71%
Delgado-Fernández et al (2022)^30^	Spain	2009–19	Retrospective observational	NA	NA	NA	Teachers	Fatal occupational traffic accident	Spanish teachers	Non-national teachers	NA	Teachers	71%
Dong et al (2009)^31^	USA	2003–06	Retrospective cohort	396 deaths; 7 200 000 at risk (FTE workers)§	103 deaths; 2 512 195 at risk (FTE workers)§	496 (99·5%); 3 (0·5%)	Hispanic construction workers aged ≥16 years	Work-related fatal fall	US-born Hispanic workers	Foreign-born Hispanic workers	Mexico, central America, South America, Caribbean, and others	Construction	75%
Dong et al (2013)^32^	USA	1992–2009 (detailed analysis 2003–09)	Retrospective cohort	293 deaths; 16 098 901 at risk (FTE workers)§	582 deaths; 58 787 878 at risk (FTE workers)§	869 (99·3%); 6 (0·7%)	Construction workers aged ≥16 years	Work-related fatal fall from roof	US-born workers	Foreign-born workers	NA	Construction	75%
Dong et al (2014)^33^	USA	2003–10	Retrospective cohort	236 deaths; population at risk NA	633 deaths; population at risk NA	866 (99·7%); 3 (0·3%)	Private wage-and-salary residential construction workers	Work-related fatal fall	US-born workers	Foreign-born Hispanic workers	NA	Construction	63%
Dunlavy et al (2018)^34^	Sweden	1993–2008	Retrospective cohort	NA	NA	1 096 275 (50·3%); 1 082 046 (49·7%)	Individuals aged 25–64 years	All-cause mortality	Swedish-born employed	Foreign-born employed	Any	NA	100%
Dunlavy et al (2019)^35^	Sweden	1993–2008	Retrospective cohort	NA	NA	1 096 275 (50·3%); 1 082 046 (49·7%)	Individuals aged 25–64 years	Suicide	Swedish-born employed	Foreign-born employed	Any	NA	100%
Hall and Greenman (2015)^36^	USA	2003–08	Retrospective cohort	3103 deaths; 44 056 412 at risk§	16 120 deaths; 433 917 613 at risk§	13 379 (54·8%); 11 023 (45·2%)	Low-wage labour force aged 18–64 years who had no more than a high school education	Fatal occupational injury	US-born workers (non-Latino White individuals)	Foreign-born Mexican and central American migrant workers	NA	NA	63%
Jayasuriya et al (2012)^37^	Sri Lanka	2009	Retrospective observational	328 deaths; population at risk NA	None	213 (64·9%); 115 (35·1%)	Sri Lankan migrant workers who died overseas	All-cause mortality	None (indirect standardisation for standardised mortality ratio)	Migrant workers of Sri Lankan nationality	Sri Lanka	NA	71%
Johansson et al (2012)^38^	Sweden	1991–2008	Retrospective cohort	NA	NA	570 010 (51·7%); 533 503 (48·3%)	Documented migrants aged 28–47 years in 1990	All-cause mortality	Swedish employed or self-employed	Foreign-born employed or self-employed	Any	NA	88%
Lee and Cho (2019)^39^	South Korea	2007–18	Retrospective observational	686 deaths; 42 089 at risk	None	34 214 (81·3%); 7875 (18·7%)	Migrant workers of Chinese nationality whose workers' compensation claims due to occupational injuries were approved	Fatal occupational injury	Korean-Chinese migrant workers	Non-national workers	China	NA	86%
Martinez (2017)^40^	USA	1992–2014	Retrospective observational	11 009 deaths; population at risk NA	6558 deaths; population at risk NA	NA	Latino workers	Fatal occupational injury	US-born Latino workers	Foreign-born Latino workers	NA	NA	43%
Menéndez et al (2013)^41^	USA	2003–08	Retrospective cohort	513 deaths; 13 768 600 at risk	549 deaths; 53 196 200 at risk	884 (83·2%); 178 (16·8%)	Workers in the retail industry	Work-related homicide	US-born workers	Foreign-born workers	NA	Retail	75%
Menendez and Havea (2011)^42^	USA	1992–2007	Retrospective observational	10 361 deaths; population at risk NA	None	9746 (94·1%); 615 (5·9%)	Foreign-born workers with fatal occupational injury	Fatal occupational injury	None	Foreign-born workers	NA	NA	57%
Mercan et al (2022)^43^	USA	1992–2016	Retrospective cohort	Deaths NA; population at risk NA (48 227 person-years)	None	23 401 (48·5%); 24 826 (51·5%)	Immigrant workers in labour force aged ≥50 years	All-cause mortality	None	Workers with migrant background from survey data	NA	NA	88%
Orrenius and Zavodny (2009)^44^	USA	2003–05	Retrospective cohort	Deaths NA; 215 223 at risk	Deaths NA; 1 492 416 at risk	922 267 (54·0%); 785 372 (46·0%)	Employed in private sector aged ≥16 years	Industry injury fatality; occupational injury fatality	US-born workers	Foreign-born workers	NA	NA	75%
Östh (2018)^45^	Sweden	1991–2010	Retrospective cohort	NA	None	1 225 012 (59·3%); 841 593 (40·7%)	Immigrants from predominantly Muslim countries aged ≥16 years	All-cause mortality	None	Immigrants who are employed	Various Islamic countries	NA	88%
Pradhan et al (2019)^46^	Qatar	2009–17	Retrospective observational	1354 deaths; 904 249 at risk	None	NA	Nepali migrant workers	Cardiovascular death	None	Nepali migrant workers	Nepal	NA	29%
Rauscher and Myers (2016)^47^	USA	2001–12 (rates and rate ratios for 2006–12)	Retrospective cohort	27 deaths; 375 000 at risk (FTE workers)§	98 deaths; 5 764 706 at risk (FTE workers)§	113 (90·4%); 12 (9·6%)	Workers aged <18 years (fatality rates available only for those aged 15–17 years)	Fatal occupational injury	US-born employed	Foreign-born employed	Mexico, central America, and others	NA	75%
Reid et al (2016)^48^	Australia	1991–2002	Retrospective cohort	1122 deaths; population at risk NA (35 486 207 person-years)	4034 deaths; population at risk NA (100 911 153 person-years)	4426 (85·8%); 730 (14·2%)	Workers	Fatal occupational injury	Australian-born workers	Foreign-born workers	Any	NA	88%
Reid et al (2018)^49^	Australia	1940–2009	Retrospective cohort	563 deaths; 1031 at risk	1876 deaths; 3465 at risk	6500 (100%); 0	Male workers exposed to blue asbestos at Wittenoom, WA	All-cause mortality; cause-specific mortality	Australian-born or UK-born workers	Italian-born workers	Italy	Mining	63%
Rey-Merchán and López-Arquillos (2021)^50^	Spain	2009–19	Retrospective observational	306 deaths; population at risk NA	2099 deaths; population at risk NA	2109 (87·7%); 296 (12·3%)	Workers who experienced occupational traffic crashes	Fatal occupational traffic crash	Spanish workers	Non-national workers	NA	NA	71%
Salem et al (2013)^51^	United Arab Emirates	2005–09	Retrospective observational	4 deaths; population at risk NA	No deaths; population at risk NA	4 (100%); 0	Patients admitted to hospital for traumatic brain injury	Occupational traumatic brain injury deaths	None	Non-national workers	NA	NA	43%
Saunders et al (2019)^52^	Canada	2003–17	Retrospective cohort	46 deaths; 243 099 at risk	None	4851 (74·8%); 1633 (25·2%)	Adults aged ≥18 years living in Ontario and eligible for provincial health care	Suicide	Long-term residents and immigrants on other visa types	Immigrants with business or economic visas	Any	NA	100%
Steege et al (2014)^53^	USA	2005–09	Retrospective cohort	4665 deaths; 116 625 000 at risk§	22 331 deaths; 8 039 160 000 at risk§	24 995 (92·6%); 2001 (7·4%)	Workers employed in high-risk occupations aged ≥16 years	Fatal occupational injury; occupational homicide	US-born workers	Foreign-born workers	NA	NA	75%
Syse et al (2018)^54^	Norway	1990–2015	Retrospective cohort	846 deaths; population at risk NA (1 179 253 person-years)	None	298 473 (60·6%); 193 698 (39·4%)	Immigrant and local workers aged 25–79 years	All-cause mortality	Norwegian-born (both workers and non-workers)	Labour migrants	NA	NA	88%
Tiagi (2015)^55^	Canada	2011	Retrospective cohort	Deaths NA; 162 710 at risk	Deaths NA; 267 858 at risk	345 086 (48·6%); 364 363 (51·4%)	Immigrant workers and local workers aged ≥15 years	Occupational fatality; industry fatality	Canadian-born workers	Foreign-born workers	NA	NA	75%
Tiagi (2016)^56^	Canada	2011	Retrospective cohort	Deaths NA; 164 320 at risk	Deaths NA; 544 903 at risk	78 050 (47·5%); 86 338 (52·5%)	Immigrants aged ≥16 years	Occupational fatality	Second-generation and third-generation immigrant workers	First-generation immigrant workers	NA	NA	63%
Uzun et al (2009)^57^	Türkiye	1998–2002	Retrospective observational	146 deaths; population at risk NA	None	113 (77·4%); 33 (22·6%)	Foreigners who died in Istanbul	All-cause mortality	Foreigners who came to visit	Foreigners who came to work	Multiple	NA	57%
Vanthomme and Gadeyne (2019)^58^	Belgium	2001–11	Retrospective cohort	Deaths NA; 300 175 at risk	Deaths NA; 2 576 554 at risk	1 616 949 (56·2%); 1 259 780 (43·8%)	Healthy Belgian population aged 25–59 years	All-cause mortality; cause-specific mortality	Local workers	Non-local workers	NA	NA	88%
Xiang et al (2020)^59^	Australia	2000–14	Retrospective cohort	59 deaths; population at risk NA	191 deaths; population at risk NA	233 (93·2%); 17 (6·8%)	Workers with accepted compensation claims in South Australia	Fatal occupational injury	Australian-born workers	Foreign-born workers	Any	NA	75%
Yamaguchi et al (2023)^60^	Japan	2011–20	Retrospective observational	13 deaths; population at risk NA	123 deaths; population at risk NA	134 (98·5%); 2 (1·5%)	Deaths with forensic autopsies in Tokyo and Chiba prefectures	Occupational accidental injury death	Japanese-born workers	Foreign-born workers	NA	NA	43%
Zheng and Yu (2022)^61^	USA	1992–2011	Retrospective cohort	Deaths NA; 73 727 at risk	Deaths NA; 410 348 at risk	257 605 (53·2%); 226 470 (46·8%)	Individuals aged 30–65 years in the labour force	All-cause mortality	US-born workers	Foreign-born workers	NA	NA	50%

NA=not available or not specified. FTE=full-time equivalent.

* Some studies reported sex and others reported gender.

† Quality of included studies was assessed using an adapted version of the Newcastle–Ottawa Scale; studies were deemed low quality if they scored <50%.

‡ Other gender: 3 (2·7%).

§ Back calculated with numerator, denominator, or rate reported in the article.

The overall quality of studies was high, with a median score of 75% (IQR 60–87). Only six studies were low quality (score of <50%), with scores ranging from 29% to 100% (table). Detailed scoring is provided in the appendix (pp 3–5). Funnel plots (appendix p 16) showed no evidence of publication bias, although patterns might not be informative due to the small number of studies available.

**Figure 2:**
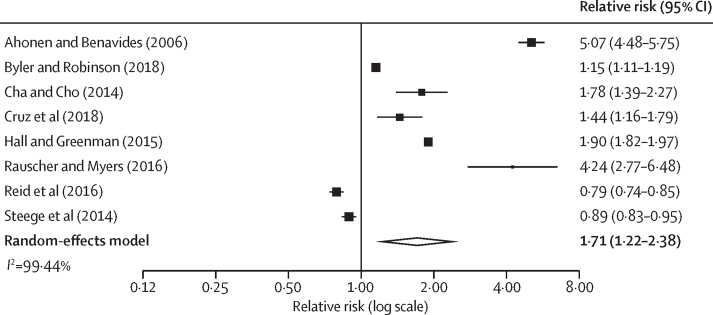
Forest plot of relative risk of fatal occupational injuries in migrant workers compared with local workers

The most reported mortality outcome was fatal occupational injury. Injuries included fall from heights, electric shocks, and being struck by objects. All 18 studies reporting fatal occupational injuries were conducted in high-income countries, and key sectors involved were agriculture, manufacturing, and construction^18^, ^19^, ^21^, ^22^, ^23^, ^24^, ^27^, ^29^, ^36^, ^39^, ^40^, ^42^, ^44^, ^47^, ^48^, ^53^, ^59^, ^60^ (appendix pp 6–7). Eight studies were considered sufficiently homogeneous based on characteristics of the migrant worker population (including sex or gender and sector) to be combined using meta-analysis^18^, ^22^, ^23^, ^27^, ^36^, ^47^, ^48^, ^53^ (figure 2). The pooled relative risk was 1·71 (95% CI 1·22–2·38), indicating that migrant workers had a higher risk of death from occupational injury compared with local workers, despite substantial statistical heterogeneity (*I*^2^=99·4%). The two studies with relative risk of less than one were an Australian study in which migrant workers were more likely to be highly skilled^48^ and a US study that included workers in only high-risk occupations and thus was already stratified on this variable.^53^ Sensitivity analyses were done in which low-quality studies were excluded or a fixed-effects model was used. Both analyses yielded similar results to the main analysis (appendix p 14). Another sensitivity analysis was done in which both low-quality and medium-quality studies were excluded. This analysis yielded a similar risk estimate, but it no longer reached statistical significance (appendix p 15).

**Figure 3:**
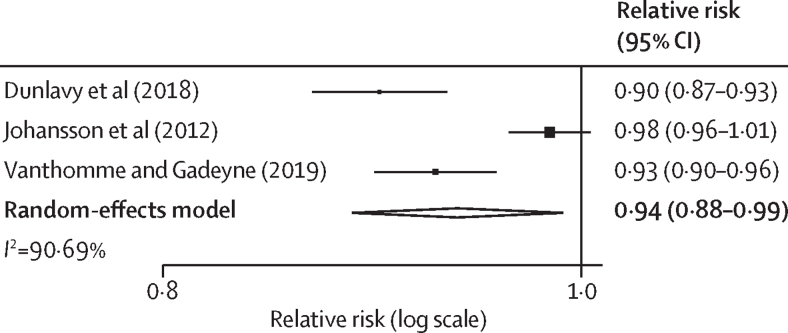
Forest plot of relative risk of all-cause mortality in migrant workers compared with local workers

The second most reported mortality outcome was all-cause mortality. 12 studies reported all-cause mortality, of which two were conducted in middle-income countries and the other ten were in high-income countries^20^, ^26^, ^34^, ^37^, ^38^, ^43^, ^45^, ^49^, ^54^, ^57^, ^58^, ^61^ (appendix pp 8–9). Three studies were deemed sufficiently homogeneous to be included in meta-analysis^34^, ^38^, ^58^ (figure 3). These studies all used country of birth to determine workers’ migrant status and all risk estimates were adjusted for key confounders (eg, age and education). The pooled relative risk was 0·94 (95% CI 0·88–0·99), indicating that migrant workers had an overall all-cause mortality advantage over local workers, despite moderate statistical heterogeneity (*I*^2^=90·7%). A sensitivity analysis was done in which a fixed-effects model was used instead of a random-effects model, and the results remained similar to the main analysis (appendix p 15).

19 studies reported other mortality outcomes, all of which were conducted in high-income countries^20^, ^24^, ^25^, ^27^, ^28^, ^30^, ^31^, ^32^, ^33^, ^35^, ^41^, ^46^, ^49^, ^50^, ^51^, ^52^, ^53^, ^55^, ^56^ (appendix pp 10–12). Migrant workers were more likely to die from external causes of death (such as falls or assaults) than internal causes of death (such as respiratory diseases or digestive diseases) compared with local workers.^20^, ^27^ Migrant workers were more likely to die from work-related homicides, especially in the retail and sex industries.^28^, ^41^, ^53^ There was some evidence of higher suicide risk in migrant workers, particularly among female migrant workers compared with female local workers.^25^, ^35^ Studies also reported higher risks for fatal occupational traffic accidents^30^, ^50^ and falls,^31^, ^32^, ^33^ with inconsistent findings for occupational diseases.^24^, ^55^, ^56^

**Figure 4:**
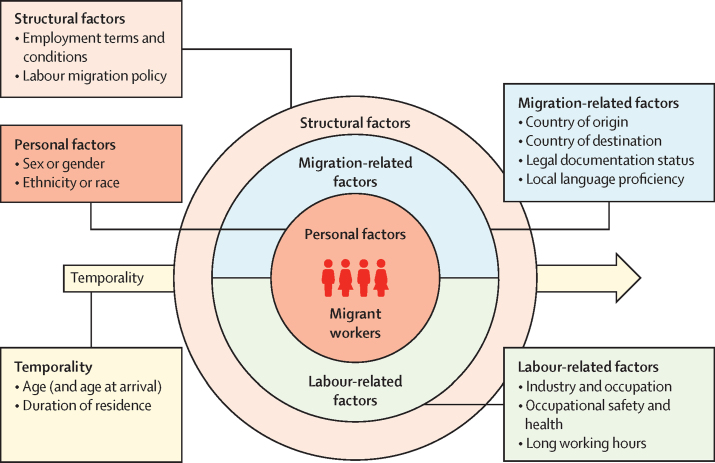
Conceptual diagram of the intersectional social determinants of mortality in migrant workers

We analysed studies by risk factor and developed a data-driven conceptual diagram summarising the intersectional social determinants of mortality (figure 4). The determinants were at the personal or structural level, migration-related or labour-related, or had a temporal dimension. The concentric multi-layered circles and the arrow underneath are intended to capture the intersecting nature of these determinants.

20 studies reported mortality risk disaggregated by sex or gender.^18^, ^25^, ^26^, ^27^, ^34^, ^35^, ^36^, ^37^, ^38^, ^39^, ^43^, ^45^, ^48^, ^53^, ^54^, ^55^, ^56^, ^58^, ^61^ Compared with local workers, male migrant workers had a higher relative risk for all-cause mortality than female migrant workers, whereas female migrant workers had a higher relative risk for other mortality outcomes (appendix p 13). By contrast, absolute risk showed a different mortality pattern, in which absolute rates of fatal occupational injury in male migrant workers were consistently higher than in female migrant workers (appendix p 13).

Four studies stratified findings by ethnicity or race, all of which were conducted in the USA.^29^, ^31^, ^41^, ^61^ Within the same ethnic or racial group, migrant workers usually had higher relative mortality risks than their local counterparts, including occupational homicide, fatal occupational injury, and fatal falls.^29^, ^31^, ^41^ Across different ethnic or racial groups, the relative mortality risk of migrant workers varied widely, up to several fold.^29^, ^61^ One study found that migrant status was likely to be a more important determinant of mortality than ethnicity after controlling for the effect of the other.^31^

Migrant workers were usually younger than local workers. When disaggregated by age, risk for fatal occupational injury was highest for the youngest and oldest migrant workers, both in terms of absolute risk and relative risk.^18^, ^47^, ^53^ Reasons included scarcity of safety training for newly arrived young migrant workers, and complacency among older migrant workers.^21^, ^31^ Age at arrival was also identified as a determinant of mortality, with evidence of higher mortality risk in migrant workers arriving at younger ages.^35^, ^54^

Mortality risk was found to be dependent on the relational positions of countries of origin and destination, such as economic disparity or cultural difference. In the USA, migrant workers from Latin America, Africa, and Asia had a higher risk of fatal occupational injury than local workers, whereas those from Europe had no risk difference.^22^ In Sweden, migrant workers from Nordic countries had a higher risk of all-cause mortality and suicide than local workers, whereas those from non-Nordic countries generally had lower risks.^34^, ^35^, ^38^ One study suggested that societal and cultural differences towards suicide could partly explain the different suicide rates among migrant workers.^25^

Legal status was an important yet understudied factor. Most studies did not distinguish between temporary migrants and permanent migrants. A US study found that undocumented migrant workers had higher rates of fatal occupational injury compared with both documented migrant workers and US-born workers.^36^ Another Turkish study found that 94·5% of foreigners who came to work and died did not have a work permit.^57^ When visa status was considered, studies from Canada and Norway found that migrants on economic or business visas had lower mortality than migrants on refugee visas and local residents.^52^, ^54^

Five studies looked at migrant workers’ language proficiency.^29^, ^36^, ^44^, ^56^, ^60^ Across the countries studied, migrant workers with lower proficiency of the local language were consistently found to have higher rates of occupational fatality. Proposed mediating pathways include difficulties in understanding safety training, bargaining for their rights at work, and following rules and regulations, and reduced job opportunities.^36^, ^60^ Language ability was also found to partly explain undocumented migrant workers’ higher occupational risk.^36^

Duration of residence was found to be associated with mortality. Although there is a general positive association between longer duration of residence and higher mortality risk, patterns differed across types of mortality outcome, sex or gender, and region of origin. In Sweden, female migrant workers with shorter residence had a lower all-cause mortality risk, whereas those with longer residence had a higher suicide risk.^35^, ^38^ In Norway, there was some evidence that migrants on labour visas with shorter residency had lower all-cause mortality risk than those with longer residency.^54^ Only one study specifically looked at return migration and found that mortality risk did not differ between migrant workers who stayed or returned.^49^

The riskiest industries for occupational fatalities were construction, agriculture, and mining, whereas the riskiest occupations were farmers, transportation workers, and machine workers.^24^, ^40^, ^53^ Ten studies focused on selected industries, namely construction,^20^, ^31^, ^32^, ^33^ agriculture,^21^, ^26^ retail,^41^ mining,^49^ sex work,^28^ and education.^30^ Migrant construction workers had higher rates of fatal falls than local workers, but lower rates of all-cause mortality.^20^, ^31^, ^32^ Over time, one study found that the risk of fatal injuries was declining for local workers but increasing for migrant workers.^24^ Studies in the USA and Qatar found that higher rates of fatal occupational injuries in migrant workers could be explained by the higher proportion of migrants working in low-skilled occupations.^19^, ^29^ When stratified by industry and by occupation, the occupational fatality rate was higher for migrant workers than local workers in some industries and occupations but not others.^24^, ^40^, ^53^ Studies have found that, after taking into account their occupations, migrant workers still had a higher fatal occupational injury risk than local workers.^22^, ^36^

Although there is evidence that occupational safety and health measures had been improving among local workers in the 2000s, they did not improve as much for migrant workers.^24^ Studies pointed to the roles of both employers and migrant workers. For employers, this included a scarcity of language-appropriate and culture-appropriate training and a scarcity of health protection measures, especially in the construction industry.^31^, ^46^ For migrants, this included non-awareness of safety guidelines and lower adherence to occupational safety practices, particularly among those with lower education.^20^, ^27^

Although there was some evidence on the risk of long working hours for mortality, this risk did not seem to be higher in migrants than among the general working population. A Singaporean study suggested that the long working hours and fatigue accumulated over the week might have resulted in higher injury rates at the end of the week.^23^ A US study found that migrants who worked long hours had no higher mortality risk than other workers who worked long hours.^43^

Studies pointed to upstream regulatory contexts that shaped the employment terms and conditions, particularly neoliberal deregulation policies, which weaken safety and health protection for workers and promote flexible labour such as part-time and temporary employment.^40^ Such regulatory changes encouraged employers to rely on subcontracting rather than hiring migrant workers and reduced employer incentives to provide safety training to temporary migrant workers.^36^ In turn, these circumstances exposed migrant workers to greater risks of occupational injuries and death due to their disproportionate representation in riskier and less stable jobs. Evidence of labour exploitation in migrant workers, including physical and emotional abuse, were also found to be contributing triggers for suicide.^25^

Labour migration policies in destination countries were found to partly explain variations in migrant worker mortality risk across countries. Labour migration policies that encouraged high skilled migration, such as in Australia and Canada, were found to be associated with lower fatal injury and suicide rates in migrant workers.^48^, ^52^ In South Korea, the policy on prohibition of changing workplaces was found to increase the risk of fatal occupational injury among migrant workers.^39^

## Discussion

To our knowledge, this study provides the strongest evidence to date of the higher fatal injury risks associated with being a migrant worker. Indeed, migrant workers have nearly twice the risk of a workplace fatal injury compared with local workers. Although migrant workers have an overall mortality advantage compared with local workers (relative risk 0·94, 95% CI 0·88–0·99), they are more likely to die from occupational injuries (1·71, 1·22–2·38), implying that opportunities exist to redress this health inequity through changing the work environment. By summarising the scientific literature to date, this global study contributes objective evidence to recent debates on whether migrant workers are at higher risk of death than local workers, sparked by media reports related to the Qatar FIFA World Cup constructions.^62^ Our study shows that migrants experience health inequities in terms of fatal occupational injury, and this is where interventions should be directed. Unfortunately, only 10% of countries globally have reported any data on migrant-disaggregated occupational injury rate according to SDG indicator 8.8.1. The underlying principle of this indicator is that countries must monitor and redress any health inequities that exist between migrant and local workers at the workplace. Our study reaffirms that work-related injuries are indeed informative indicators to monitor health inequalities in migrants, and propose that this indicator should extend beyond 2030 to ensure countries continue to collect and report on such data, and are held accountable to actions taken towards reducing this health inequity. Efforts should also be strengthened to collect and report data on occupational diseases disaggregated by migrant status, particularly due to long latency periods when migrants might have left the country by the time they develop symptoms or are diagnosed.

Migrants’ increased risk of workplace fatality could be explained by reasons including higher exposure to dangerous and unsafe environments, inadequate labour and social protection, and barriers in accessing health services due to founded or unfounded worries of detention by the authorities, among others.^4^ Our findings add to this body of knowledge by explicitly acknowledging the roles of structural determinants of health, particularly delineating risk factors that are related to being a migrant, being a worker, and those exacerbated because of being a worker who is also a migrant. It is important to acknowledge the root causes for which people migrate to seek work that place them at higher risk of injury and death by injury, including economic disparity between sending and receiving countries, and labour shortages in high-income settings due to ageing populations and local workers’ reluctance to partake in low-skilled jobs.^63^ We proposed a data-driven framework to explicate the intersectionality between migration-related and labour-related factors at the structural level, along a temporal dimension corresponding to the stages of migration now considered fundamental to migration and health research.^64^ Our emphasis on the intersectional nature of risk factors is intended to encourage researchers and practitioners to take this into consideration when designing policies and interventions. For example, preventive measures at the individual level, such as providing occupational safety training to migrants, should not only take into consideration language and cultural sensitivities, but also the fact that new arrivals are often unaware of their legal rights and should therefore be given additional information and support compared with local workers. Preventive measures at the structural level could include extending labour protection laws and compensation mechanisms to migrant workers, because many countries continue to exclude non-citizens in social protection legislations, contrary to the principle of equality of treatment advocated by the ILO for migrant workers in national labour and social protection legislations.^65^ Other policy interventions include ensuring that labour inspectorates have a primary duty to safeguard the work rights of migrant workers instead of enforcing immigration law, and offering legal protection against retaliation or deportation to migrant workers who report on employers’ labour rights violations. Our hope is that readers would begin shifting their attention from interventions that target individual-level determinants to those that address more upstream, structural-level determinants of migrant worker health.

This study has several key limitations. Because most of the included studies were conducted in high-income settings, findings might not be generalisable to migrant workers in low-income and middle-income countries (LMICs), where there are more informal jobs, fewer workplace protections, and weaker regulations. Mortality estimates might therefore be far higher in LMICs and there is an urgent need for more research in these settings. Moreover, most studies relied on either population registries or administrative records as sources of data. Although studies using national or local records were able to capture a representative sample of registered migrant workers, selection bias exists where hard-to-reach migrant populations are probably excluded (such as irregular migrants and temporary migrants) who are often at greater risk of health harms. Thus, findings from this study are necessarily an underestimation of mortality risk and should be interpreted as lower bound estimates. Returning migrants are an important group to consider because of the so-called salmon bias effect, where migrants who become ill at older ages return to their countries of origin.^66^ Moreover, we were not able to distinguish between temporary migrants and permanent migrants in our analysis. Permanent migrants are likely to have advantages over temporary migrants, such as better labour protection and access to care, thus the worse health outcomes among temporary migrants could remain hidden. In addition, substantial heterogeneity remains among studies included in meta-analyses that were deemed adequately homogeneous with respect to clinical and methodological heterogeneity. This is explained in part by differences in sociodemographic characteristics, as found in subgroup analyses, such as sex or gender and age variations, but it also reflects the variations observed across country contexts inherent in migrant health research. When subgroup analyses by sex or gender and age group were conducted, the *I*^2^ statistic was slightly reduced but remained above 90%, indicating that substantial residual heterogeneity remains. We also attempted to conduct meta-regression by age, but findings were inconsistent and therefore not reported. Although these data limitations are common in migrant health research and not only in migrant worker health research, an additional challenge is the scarcity of data linkage between migration, work, and health. Nevertheless, a strength of this study is the focus of mortality of migrant workers, because it is a more reliable estimate than morbidity outcomes due to lower likelihood of under-reporting. Although reporting bias is minimised due to the majority of studies using existing records to ascertain mortality outcomes, the overall risk of bias of this systematic review is still considered moderate, because of the high risk of selection bias due to exclusion of at-risk migrant groups not captured in these records. Nevertheless, the majority of included studies were of high or medium quality, and the exclusion of low-quality studies did not affect study findings.

Our findings suggest that much more needs to be done to reduce injuries and deaths in migrant workers. Future research must address the data gaps between work, health, and migration by establishing integrated data collection mechanisms that enable countries to monitor the health needs, injuries, illnesses, and deaths of migrant workers. Understudied determinants of health among migrant workers, including employment terms and conditions, should be a research priority. Longitudinal administrative data available in many high-income settings could be leveraged to clarify the causal pathways between work exposures and health outcomes in migrants. Data and methodological innovation will be crucial in conducting migrant worker health research in LMIC settings, as well as in identifying hard-to-reach migrant populations, such as those in informal sectors and temporary work. Only one included study was related to heat stress;^46^ future studies should respond to new and changing forms of work, including climate change and platform economy. An interesting research question is whether population-based or risk-based interventions^67^ might be more effective in protecting migrant workers. Considering that migrants are over-represented in high-risk sectors and occupations, interventions that improve the occupational safety and health of all workers in these sectors and occupations might present a less politically charged approach to safeguard the wellbeing of migrant workers. Globally, labour migration continues to fill key workforce shortages and sustains the local economy. State and private actors have both a moral and legal responsibility to prevent avoidable work-related deaths and injuries in this essential workforce. With the recent elevation of occupational safety and health as a fundamental principle and right at work,^68^ now is the opportune time to realise the right to a safe and healthy working environment for all workers, including migrants.

### Contributors

### Data sharing

The study protocol is available on PROSPERO at https://www.crd.york.ac.uk/prospero/display_record.php?RecordID=372893. Data files are available in City St George's data repository (https://doi.org/10.24376/rd.sgul.26939833.v1). Additional data access requests can be emailed to the corresponding author.

## Declaration of interests

We declare no competing interests.
