# Supplementary material for: Workplace mortality risk and social determinants among migrant workers: a systematic review and meta-analysis
Source: Lancet Public Health. 2019 Jun 17;9(11):e935–49. doi: 10.1016/S2468-2667(24)00226-3 (PMC11868777; doi:10.1016/S2468-2667(24)00226-3)
Supplement: Supplementary appendix [file mmc1.pdf]

THE LANCET
Public Health

Supplementary appendix

This appendix formed part of the original submission and has been peer reviewed. We post it as supplied by the authors.

Supplement to: Lau K, Aldridge R, Norredam M, et al. Workplace mortality risk and social determinants among migrant workers: a systematic review and meta-analysis. *Lancet Public Health* 2024; **9:** e935–49.

Supplementary Material

Workplace mortality risk and social determinants among migrant workers: a systematic review and meta-analysis

Karen Lau, MSc, Robert W Aldridge, PhD, Marie Norredam, PhD, George Frederick Mkoma, PhD, Mathura Kugan, MSc, Rosita Chai-Lin, MSc, Ligia Kiss*, Cathy Zimmerman, PhD*, Sally Hargreaves, PhD*

*Joint senior authors

S1. Example of search strategy used in Ovid MEDLINE

1 exp "Transients and Migrants"/
2 exp "Emigrants and Immigrants"/
3 exp Human Migration/
4 exp Refugees/
5 asyl*.mp.
6 emigra*.mp.
7 immigra*.mp.
8 expat*.mp.
9 foreign*.mp.
10 migrant*.mp.
11 oversea*.mp.
12 refuge*.mp.
13 traffick*.mp.
14 undocument*.mp.
15 1 or 2 or 3 or 4 or 5 or 6 or 7 or 8 or 9 or 10 or 11 or 12 or 13 or 14
16 exp Working Poor/
17 blue collar.mp.
18 employ*.mp.
19 labor*.mp.
20 labour*.mp.
21 slave*.mp.
22 worker*.mp.
23 working*.mp.
24 work*.mp.
25 wage*.mp.
26 exp Income/
27 exp Employment/
28 exploit*.mp.
29 16 or 17 or 18 or 19 or 20 or 21 or 22 or 23 or 24 or 25 or 26 or 27 or 28
30 exp Death/
31 exp Mortality/
32 exp Life Expectancy/
33 exp Suicide/
34 exp Homicide/
35 dead.mp.
36 death*.mp.

37 die.mp.
38 dying.mp.
39 fatal*.mp.
40 exp Longevity/
41 SMR.mp.
42 mortalit*.mp.
43 homicid*.mp.
44 life expectanc*.mp.
45 suicid*.mp.
46 longevit*.mp.
47 30 or 31 or 32 or 33 or 34 or 35 or 36 or 37 or 38 or 39 or 40 or 41 or 42 or 43 or 44 or 45 or 46
48 exp Cells/
49 exp Animal Migration/
50 exp Proteins/
51 exp Tissues/
52 exp Genes/
53 exp In Vitro Techniques/
54 vivo.mp.
55 exp Laboratories/
56 exp animals/ not humans.sh.
57 48 or 49 or 50 or 51 or 52 or 53 or 54 or 55 or 56
58 15 and 29 and 47
59 58 not 57
60 limit 59 to (english language and yr="2000 -Current")

S2. Risk of bias assessment using the adapted version of the Newcastle Ottawa Scale

Cohort studies

	Selection											Comparability	
	Representativeness of the exposed cohort/sample				Selection of the non-exposed cohort			Ascertainment of exposure				Comparability on the basis of the design or analysis controlled for confounders	
	Truly representative (one star)	Somewhat representative (one star)	Selected group (no star)	No description of the derivation of the cohort/sampling strategy (no star)	Drawn from the same community as the exposed cohort (one star)	Drawn from a different source (no star)	No description of the derivation of the non-exposed cohort (no star)	Secure record (one star)	Structured interview (one star)	Written self report (no star)	No description (no star)	Study controls for relevant factors (e.g. age, sex) (one star)	Not comparable on the basis of study design or analysis (no star)
**Ahonen & Benavides (2006)**		1			1			1				1	
**Al-Thani et al. (2015)**	1				1			1					0
**Arndt et al. (2004)**		1			1						0	1	
**Byler & Robinson (2018)**	1				1			1				1	
**Carangan, M., et al. (2004)**			0		1				1				0
**Cha & Cho (2014)**	1				1			1				1	
**Cooper et al. (2001)**			0		1				1				0
**Cruz et al. (2018)**	1					0		1					0
**Dong et al. (2009)**	1				1			1					0
**Dong et al. (2013)**	1				1			1					0
**Dong et al. (2014)**	1				1			1					0
**Dunlavy et al. (2018)**	1				1			1				1	
**Dunlavy et al. (2019)**	1				1			1				1	
**Hall & Greenman (2015)**	1				1				1				0
**Johansson et al. (2012)**	1				1			1				1	
**Menendez et al. (2013)**	1				1			1				1	
**Mercan et al. (2022)**		1			1				1			1	
**Orrenius & Zavodny (2009)**		1			1				1			1	
**Osth (2018)**	1				1			1				1	
**Rauscher & Myers (2016)**	1				1			1					0
**Reid et al. (2016)**	1				1			1				1	
**Reid et al. (2018)**			0		1					0		1	
**Saunders et al. (2019)**	1				1				1			1	
**Steege et al. (2014)**	1				1			1				1	
**Syse et al. (2018)**	1				1			1				1	
**Tiagi (2015)**		1			1			1				1	
**Tiagi (2016)**		1			1			1					0
**Vanthomme & Gadeyne (2019)**	1				1			1				1	
**Xiang et al. (2020)**		1			1			1					0
**Zheng & Yu (2022)**	1				1			1					0

	Outcome													Total score		
	Assessment of outcome					Was duration of follow up explicitly indicated		Adequacy of follow-up cohorts				Statistical test		Num-erator	Denom-inator (n=8)	Per-centage
	Independent blind assessment (one star)	Record linkage (one star)	Self report (no star)	No description (no star)	Other (no star)	Yes (one star)	No (no star)	Complete follow up reported, all subjects accounted for (one star)	Subjects lost to follow-up are discussed or are unlikely to introduce bias (one star)	Subjects lost to follow-up are not discussed or may introduce bias (no star)	No reporting of subjects lost to follow-up (no star)	Are sufficient data presented to support the estimates or conclusions drawn (measures of precision reported, denominators reported) (one star)	The statistical test is not appropriate, not described or incomplete (no star)			
**Ahonen & Benavides (2006)**		1					0				0	1		6	8	75%
**Al-Thani et al. (2015)**		1				1					0	1		6	8	75%
**Arndt et al. (2004)**	1					1			1			1		7	8	88%
**Byler & Robinson (2018)**		1				1					0	1		7	8	88%
**Carangan, M., et al. (2004)**		1				1				0			0	4	8	50%
**Cha & Cho (2014)**		1					0			0		1		6	8	75%
**Cooper et al. (2001)**		1				1			1				0	5	8	63%
**Cruz et al. (2018)**		1					0			0			0	3	8	38%
**Dong et al. (2009)**		1				1					0	1		6	8	75%
**Dong et al. (2013)**		1				1					0	1		6	8	75%
**Dong et al. (2014)**		1				1					0		0	5	8	63%
**Dunlavy et al. (2018)**		1				1			1			1		8	8	100%
**Dunlavy et al. (2019)**		1				1			1			1		8	8	100%
**Hall & Greenman (2015)**		1				1					0		0	5	8	63%
**Johansson et al. (2012)**		1				1					0	1		7	8	88%
**Menendez et al. (2013)**		1					0				0	1		6	8	75%
**Mercan et al. (2022)**				0		1		1				1		7	8	88%
**Orrenius & Zavodny (2009)**		1					0				0	1		6	8	75%
**Osth (2018)**		1				1					0	1		7	8	88%
**Rauscher & Myers (2016)**		1				1					0	1		6	8	75%
**Reid et al. (2016)**		1				1					0	1		7	8	88%
**Reid et al. (2018)**	1					1					0	1		5	8	63%
**Saunders et al. (2019)**		1				1			1			1		8	8	100%
**Steege et al. (2014)**		1					0				0	1		6	8	75%
**Syse et al. (2018)**		1				1				0		1		7	8	88%
**Tiagi (2015)**		1					0				0	1		6	8	75%
**Tiagi (2016)**		1					0				0	1		5	8	63%
**Vanthomme & Gadeyne (2019)**		1				1					0	1		7	8	88%
**Xiang et al. (2020)**		1				1			1				0	6	8	75%
**Zheng & Yu (2022)**		1					0				0		0	4	8	50%

Other observational studies

	Selection											
	Representativeness of the exposed cohort/sample				Sample size		Non-respondents			Ascertainment of the exposure (risk factor)		
	Truly representative (one star)	Somewhat representative (one star)	Selected group (no star)	No description of the derivation of the cohort/sampling strategy (no star)	Justified and satisfactory (consider statistical power and transparency of reporting) (one star)	Not justified (no star)	Comparability between respondents and non-respondents characteristics is established, and the response rate is satisfactory (one star)	The response rate is unsatisfactory, or the comparability between respondents and non-respondents is unsatisfactory (no star)	No description of the response rate or the characteristics of the respondents and non-respondents (no star)	Validated measurement tool (one star)	Non-validated measurement tool, but the tool is available or described (one star)	No description of the measurement tool (no star)
**Baraza & Cuguero-Escofet (2022)**	1					0	1			1		
**Chiu et al. (2022)**		1				0	1				1	
**Cunningham et al. (2018)**		1				0			0		1	
**Davila et al. (2011)**	1					0	1				1	
**Delgado-Fern et al. (2022)**	1					0	1				1	
**Jayasuriya et al. (2012)**		1				0		0			1	
**Lee & Cho (2019)**	1					0	1			1		
**Martinez (2017)**	1					0			0	1		
**Menendez & Havea (2011)**	1					0	1			1		
**Pradhan et al. (2019)**			0			0			0		1	
**Rey-Merchan & Lopez-Arquillos (2021)**	1					0	1			1		
**Salem et al. (2013)**			0			0	1			1		
**Uzun et al. (2009)**		1				0	1				1	
**Yamaguchi et al. (2023)**			0			0	1			1		

	Comparability		Outcome							Total score		
	Comparability on the basis of the design or analysis controlled for confounders		Assessment of outcome					Statistical test		Num-erator	Denom-inator (n=7)	Percentage
	Study controls for relevant factors (e.g. age, sex) (one star)	Not comparable on the basis of study design or analysis (no star)	Independent blind assessment (one star)	Record linkage (one star)	Self report (no star)	No description (no star)	Other (no star)	Are sufficient data presented to support the estimates or conclusions drawn (measures of precision reported, denominators reported) (one star)	The statistical test is not appropriate, not described or incomplete (no star)			
**Baraza & Cuguero-Escofet (2022)**		0		1					0	4	7	57%
**Chiu et al. (2022)**		0		1				1		5	7	71%
**Cunningham et al. (2018)**		0		1					0	3	7	43%
**Davila et al. (2011)**		0		1				1		5	7	71%
**Delgado-Fern et al. (2022)**		0		1				1		5	7	71%
**Jayasuriya et al. (2012)**	1			1				1		5	7	71%
**Lee & Cho (2019)**	1			1				1		6	7	86%
**Martinez (2017)**		0		1					0	3	7	43%
**Menendez & Havea (2011)**		0		1					0	4	7	57%
**Pradhan et al. (2019)**		0	1						0	2	7	29%
**Rey-Merchan & Lopez-Arquillos (2021)**		0		1				1		5	7	71%
**Salem et al. (2013)**		0		1					0	3	7	43%
**Uzun et al. (2009)**		0		1					0	4	7	57%
**Yamaguchi et al. (2023)**		0	1	0					0	3	7	43%

S3. Included studies reporting fatal occupational injury in migrant workers.

Study	Country	Region	Mortality outcome	Measure	Key findings	Age disaggregation	Sex/gender disaggregation	Adjusted for confounders
Ahonen, E. Q. and F. G. Benavides (2006)	Spain	Europe	Fatal occupational injury	Incidence rate; Relative risk	Compared to Spanish workers, foreign workers had a relative risk of fatal occupational injury of 4.4 (95%CI: 3.9-5.1) for men and 6.0 (95%CI: 3.6-9.6) for women respectively.	Y	Y	N
Al-Thani, H., et al. (2015)	Qatar	Middle East	Fatal occupational injury	Proportion; Mean; Median; Rate; Relative risk; Mortality burden; Admitted case fatality rate	Among the nation-wide occupational injury hospital admissions during 2010-2013, there were 86 foreigner deaths, compared to zero local Qatari deaths.	N	N	N
Baraza, X. and N. Cuguero-Escofet (2022)	Spain	Europe	Fatal occupational accident	Fatal accident rate (FAR) (actually a proportion) = no. of fatal accidents in the category studied / total no. of fatal accidents	The fatal accident rate for foreign workers was 23.0% and there was a notably higher incidence of fatalities for foreign workers compared to Spanish nationals.	N	N	N
Byler, C. G. and W. Robinson (2018)	USA	North America	Fatal work injury	Proportional hazards; Hazard ratio	Compared to US-born workers, foreign-born workers had a hazard ratio for fatal occupational injury of 1.15 (95%CI: 1.11-1.19), with some variation across countries of origin.	N	N	Y (gender, race/ethnicity, occupation)
Carangan, M., et al. (2004)	Singapore	Asia	Died in hospital	Proportion	Among patients admitted to hospital emergency department for work injuries in Singapore, 3/1936 of migrant workers died, compared to 0/1244 of local workers.	N	N	N
Cha, S. and Y. Cho (2014)	South Korea	Asia	Fatal occupational injury	Rate; Rate ratio; SMR	Compared to Korean workers, migrant workers had a crude relative risk for fatal occupational injury of 1.78 (95%CI: 1.39-2.27).	Y (only for all industries - not industry-specific)	N	N
Cruz, Y., et al. (2018)	USA	North America	Fatal occupational injury	Annual fatality rate; Proportion	Foreign-born workers in Kentucky had a fatal occupational rate of 7.1 per 100,000, compared to local-born workers of 4.7 per 100,000.	N	Y	N
Davila, A., et al. (2011)	USA	North America	Occupational injury fatality	Rate	Hispanic immigrant men had a fatal occupational injury rate of 0.66 per 10,000 workers, compared to 0.51 in US-born non-Hispanic White men and 0.57 in US-born Hispanic men.	N	Y (male only)	N

Study	Country	Region	Mortality outcome	Measure	Key findings	Age disaggregation	Sex/gender disaggregation	Adjusted for confounders
Hall, M. and E. Greenman (2015)	USA	North America	Occupational injury fatality	Occupation-specific fatality rate	Low-skilled undocumented immigrant workers from Mexico and Central America had a fatal occupational injury rate of 10.68 per 100,000 in men and 4.07 per 100,000 in women, compared to 9.02 per 100,000 in US-born non-Latino white men and 2.20 per 100,000 in women.	N	Y	N
Lee, J. Y. and S. I. Cho (2019)	South Korea	Asia	Fatal occupational injury	Fatality rate; OR	Compared to Korean-Chinese migrant workers, the odds ratios for fatality rate of occupational injuries of Chinese migrant workers was 1.80 (95%CI: 1.31-2.46), where authors attributed this to the policy of prohibition of changing workplaces.	Y	Y	Y (age, sex/gender, occupation, industry, year)
Martinez, R. O. (2017)	USA	North America	Fatal work injury	Count	From 1992 to 2014, the number of fatal work injuries among Latinos was increasing, but the rates among foreign-born Latino workers were higher than those for local-born Latino workers.	N	N	N
Menendez, C. K. and S. A. Havea (2011)	USA	North America	Fatal occupational injury	Proportion	10,361 individuals reported as foreign-born who suffered a fatal traumatic injury as a result of work-related activities from 1992 to 2007.	N	N	N
Orrenius, P. M. and M. Zavodny (2009)	USA	North America	Occupational injury fatality	Industry fatality rate; Occupational fatality rate	Immigrant workers had an occupational fatality rate of 7.54 per 100,000, compared to 5.93 per 100,000 in local workers.	N	N	N
Rauscher, K. J. and D. J. Myers (2016)	USA	North America	Fatal occupational injury	Fatality rate (only for 15-17yo)	Compared to local-born adolescent workers, foreign-born adolescent workers had relative risk for fatal occupational injury of 4.35 (95%CI: 2.73-6.72).	N	N	N
Reid, A., et al. (2016)	Australia	Australasia	Death from work-related injury	Rate; Mortality rate ratios	Relative risks of fatal work-related injuries were generally lower or no different between Australian and foreign-born workers, with the exception of New Zealand-born men who had excess mortality.	Y	Y	Y (age, sex/gender)
Steege, A. L., et al. (2014)	USA	North America	Fatal occupational injury	Fatality rate; RR	Compared to local-born workers in high risk occupations, foreign-born workers in high risk occupations had an adjusted rate ratio of 0.89 (95%CI: 0.83-0.95) for fatal occupational injuries.	Y	Y	Y (sex/gender, race/ethnicity, education, wages, industry, occupation)
Xiang, J., et al. (2020)	Australia	Australasia	Death from work-related injury	Case fatality rate	Foreign-born workers had higher case fatality rates for occupational injury claims than Australian-born workers, implying that foreign-born workers are at higher risk of severe injuries.	N	N	N
Yamaguchi, R., et al. (2023)	Japan	Asia	Occupational accidental injury death	Proportion	Foreign-born workers comprised 9.6% of all occupational accidental injury deaths (OAID), which was significantly higher than 3.0% in non-OAID cases.	N	N	N

S4. Included studies reporting all-cause mortality in migrant workers.

Study	Country	Region	Mortality outcome	Measure	Key findings	Age disaggregation	Sex/gender disaggregation	Adjusted for confounders
Arndt, V., et al. (2004)	Germany	Europe	All-cause mortality	SMR	Using the Benden-Wurttemberg general population as reference, construction workers of non-German nationality had an SMR for all-cause mortality of 0.57 (95%CI: 0.49-0.67), compared to 0.75 (95%CI: 0.70-0.81) in German construction workers.	N	Y (Male only)	Y (age, sex/gender, calendar year mortality)
Cooper, S. P., et al. (2001)	USA	North America	Deaths	Proportion	From 1984-1987 to 1995-1996, among a cohort of 196 migrant farmworkers, 15 had died, and 18 were lost to follow up.	Y	Y	N
Dunlavy, A. C., et al. (2018)	Sweden	Europe	All-cause mortality	HR	Compared to local Swedish employed men and women, the adjusted hazard ratio for all-cause mortality among employed men and women from foreign origins ranged from 0.41 (95%CI 0.27-0.61) in Middle Eastern females to 1.19 (95%CI: 1.10-1.28) in Finnish males.	N	Y	Y (employment status, calendar period, education level, civil status, disposable income quintile)
Jayasuriya, V., et al. (2012)	Sri Lanka	Asia	Deaths	SMR	Using the Sri Lankan population as reference, Sri Lankan migrant workers had an SMR of 0.31 for males and 0.37 for females. The only age-sex group with a higher SMR than one was female aged 25-29 (SMR = 1.04).	Y	Y	Y (age and sex/gender)
Johansson, B., et al. (2012)	Sweden	Europe	Deaths	HR	Compared to local Swedish men and women, employed/self-employed immigrant men and women had an adjusted hazard ratio for all-cause mortality of 1.2 (95%CI: 1.2-1.2) and 1.0 (95%CI: 0.9-1.0) respectively.	Y	Y	Y (age, education, income, time since first migration, white- and blue-collar employment)
Mercan, M. A., et al. (2022)	USA	North America	All-cause mortality	HR	Working 50 hours or more per week has an adjusted hazard ratio for mortality of 1.46 (95%CI: 0.86-2.50) in immigrant workers, compared to 1.45 (95%CI: 0.86-2.45) in workers in labour force in general.	N	Y	Y (age, race/ethnicity, income, marriage, health status, number of jobs, etc)
Osth, J. (2018)	Sweden	Europe	Mortality	OR	Among migrants born in Islamic countries and residing in Sweden, men employed had a hazard ratio for mortality of 0.45 (95%CI: 0.40-0.50) compared to those outside the labour market, while women had 0.52 (0.43-0.64).	Y (age >=16 or 16-55)	Y	Y (civil status, education, migration history, calendar month of death, etc)
Reid, A., et al. (2018)	Australia	Australasia	All causes	SMR	Using Western Australian population as reference, Italian workers exposed to blue asbestos had an SMR for all-cause mortality of 1.33 (95%CI: 1.22-1.44), compared to Australian workers of 1.10 (95%CI: 1.05-1.15).	N	Y (Male only)	Y (age, sex/gender)

Study	Country	Region	Mortality outcome	Measure	Key findings	Age disaggregation	Sex/gender disaggregation	Adjusted for confounders
Syse, A., et al. (2018)	Norway	Europe	All-cause death	Rate; OR	Compared to Norwegian-born locals, labour migrants had an adjusted odds ratio for mortality of 0.39 (0.37-0.42).	N	Y	Y (age group, sex/gender, calendar period, education, parenthood, marital status)
Uzun, I., et al. (2009)	Turkey	Europe	Death	Proportion	Among 411 foreigner deaths in Istanbul, 146 were employed, of which 94.5% did not have a work permit.	N	Y	N
Vanthomme, K. and S. Gadeyne (2019)	Belgium	Europe	All-cause mortality	Age-standardized all-cause mortality rates (ASMR); Age-adjusted all-cause mortality rate ratios (MRR)	Compared to local employed Belgian men and women, non-local employed men had an adjusted all-cause mortality rate ratio of 0.92 (95%CI: 0.89-0.96), while non-local employed women had 0.94 (95%CI: 0.88-1.00)	N	Y	Y (age)
Zheng, H. and W. H. Yu (2022)	USA	North America	Death	HR	Compared to US-born employed men and women, foreign-born employed men had a mortality hazard ratio of 0.85, while foreign-born employed women had 0.78.	N	Y	N

S5. Included studies reporting other mortality outcomes in migrant workers.

Study	Country	Region	Mortality outcome	Measure	Key findings	Age disaggregation	Sex/gender disaggregation	Adjusted for confounders
Cause-specific mortality								
Arndt, V., et al. (2004)	Germany	Europe	Cause-specific mortality	SMR	Using the Benden-Wurttemberg general population as reference, construction workers of non-German nationality had lower SMRs for death due to cancer, circulatory system, respiratory system, and digestive system, compared to that in German construction workers, although not statistically significant.	N	Y (Male only)	Y (Age, sex/gender, calendar year mortality)
Cruz, Y., et al. (2018)	USA	North America	External cause of mortality	Annual fatality rate; Proportion	25% and 15% of foreign-born worker fatalities were due to falls and assaults respectively, compared to 10% and 6% in local-born workers.	N	Y	N
Pradhan, B., et al. (2019)	Qatar	Middle East	Cardiovascular death	Proportion	As many as 200 of 571 cardiovascular deaths in Nepali migrant workers could have been prevented if effective heat protection measures had been implemented.	N	N	N
Reid, A., et al. (2018)	Australia	Australasia	A number of causes of deaths; All cancers	SMR	Among various causes of death, Italian migrant workers had a higher mesothelioma mortality rate of 184 per 100,000 (95%CI: 148-229) than local Australian workers (128, 95%CI: 111-149).	N	Y (Male only)	Y (Age)
Work-related homicide								
Cunningham, S., et al. (2018)	UK	Europe	Occupational homicide	Proportion	The percentage of sex work occupational homicide victims in the UK who were migrant workers increased from 6% during 1990-1999 to 50% during 2010-2016.	N	N	N
Menendez, C. C., et al. (2013)	USA	North America	Work-related homicide	Fatality rate; Rate ratio	Compared to local-born workers, foreign-born workers in selected retail industries in the US had a rate ratio of 3.5 (95%CI: 3.1-3.9) for work-related homicides.	Y (Male only)	Y (Male only)	N
Steege, A. L., et al. (2014)	USA	North America	Occupational homicide	Fatality rate; RR	Compared to local-born workers, foreign-born workers in the US had an adjusted rate ratio of 1.81 (95%CI: 1.59-2.07) for occupational homicides.	Y	Y	Y (sex/gender, race/ethnicity, education, wages, industry, occupation)
Suicide								
Chiu, M. Y. L., et al. (2022)	Singapore	Asia	Completed suicide	Proportion	Suicide notes of non-residents and residents who completed suicides in Singapore were analysed, the percentage of workers among migrants who committed suicide was 76.6%, compared to 35.8% among locals.	Y	Y	N
Dunlavy, A. C., et al. (2019)	Sweden	Europe	Suicide	HR	Compared to local-born workers in Sweden, female migrant workers had a higher risk of suicide (aHR=1.31, 95%CI: 1.03–1.68) while male migrant workers had a lower risk (aHR=0.85, 95%CI: 0.73–0.98).	N	Y	Y (education level, civil status, disposable income quintile, follow up period)

Study	Country	Region	Mortality outcome	Measure	Key findings	Age disaggregation	Sex/gender disaggregation	Adjusted for confounders
Saunders, N. R., et al. (2019)	Canada	North America	Completed suicide	Rate; Adjusted hazard ratio	Migrants on economic or business visa in Canada had suicide rate of 2.77 per 100,000 (95%CI: 2.03-3.70), compared to 6.12 (95%CI: 3.88-9.18) for refugee visas and 3.26 (95%CI:2.50-4.19) for family visas.	N	N	Y (age, sex/gender, neighbourhood income, rurality)
Specific types of fatal occupational injury								
Fatal occupational traffic accident								
Delgado-Fern, et al. (2022)	Spain	Europe	Fatal occupational traffic accident	OR	Compared to foreign teachers, Spanish teachers had an odds ratio for fatal occupational traffic accidents of 0.75 (95%CI: 0.1-5.6).	N	Y (Male only)	N
Rey-Merchan, M. D. C. and A. Lopez-Arquillos (2021)	Spain	Europe	Fatal occupational traffic crash	OR	Compared to foreign workers, Spanish workers had an odds ratio for fatal occupational traffic injuries of 0.60 (95%CI: 0.53-0.67).	N	N	N
Fatal occupational fall								
Dong, X. S., et al. (2009)	USA	North America	Work-related fatal fall	Rate; OR	Among Hispanic construction workers in the US, foreign-born workers had an odds ratio for fatal falls of 1.36 (95%CI: 1.08-1.67) compared to US-born workers.	N	N	N
Dong, X. S., et al. (2013)	USA	North America	Work-related fatal fall from roof	Rate	Rate of fatal falls from roofs in foreign-born construction workers in the US was 1.82 per 100,000 (95%CI: 1.79-1.84) full-time equivalent workers (FTE), compared to 0.99 (95%CI: 0.98-0.99) in local-born worker construction workers.	N	N	N
Dong, X. S., et al. (2014)	USA	North America	Work-related fatal fall	Proportion	27.2% of fall fatalities in residential construction workers in the US were foreign-born, compared to 23.0% in non-residential construction workers (p<0.001).	N	N	N
Fatal occupational traumatic brain injury								
Salem, A. M., et al. (2013)	UAE	Middle East	Occupational traumatic brain injury deaths	Proportion	All cases of occupational traumatic brain injuries in Abu Dhabi were male migrants, of which 8% resulted in deaths.	N	Y (Male only)	N
Fatal occupational disease								
Cha, S. and Y. Cho (2014)	South Korea	Asia	Fatal occupational disease	Rate; Rate ratio; SMR	Compared to Korean workers, migrant workers had a crude relative risk for fatal occupational disease of 0.72 (95%CI: 0.46-1.12).	Y	N	Y (Age)

Study	Country	Region	Mortality outcome	Measure	Key findings	Age disaggregation	Sex/gender disaggregation	Adjusted for confounders
Tiagi, R. (2015)	Canada	North America	Occupational fatality; Industry fatality	Rate	Compared to Canadian born men and women workers, foreign-born men workers had a lower occupational fatality rate and foreign-born women workers had a higher occupational fatality rate, although none were statistically significant.	N	Y	Y (age, self-employment, civil status, education, language, race/ethnicity)
Tiagi, R. (2016)	Canada	North America	Occupational fatality	Incidence rate ratio	Compared to second generation immigrants in Canada, first generation male immigrants had a rate ratio for occupational fatality of 1.07 (95%CI: 1.05–1.10), while first generation female immigrants had a rate ratio of 1.12 (95%CI: 1.10–1.14).	N	Y	Y (age, self-employment, civil status, education, language, race/ethnicity)

S6. Relative risk of mortality outcomes in migrant workers compared to local workers, by sex/gender.

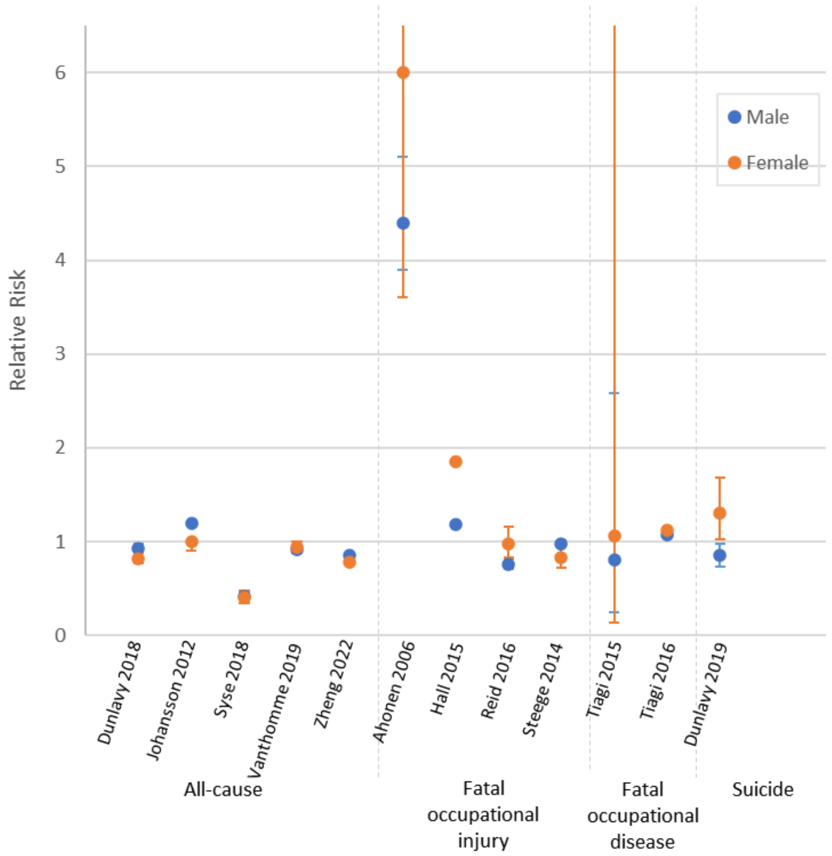

*error bars are 95% confidence intervals, where available.

S7. Absolute risk and relative risk of fatal occupational injury in migrant workers, by sex/gender.

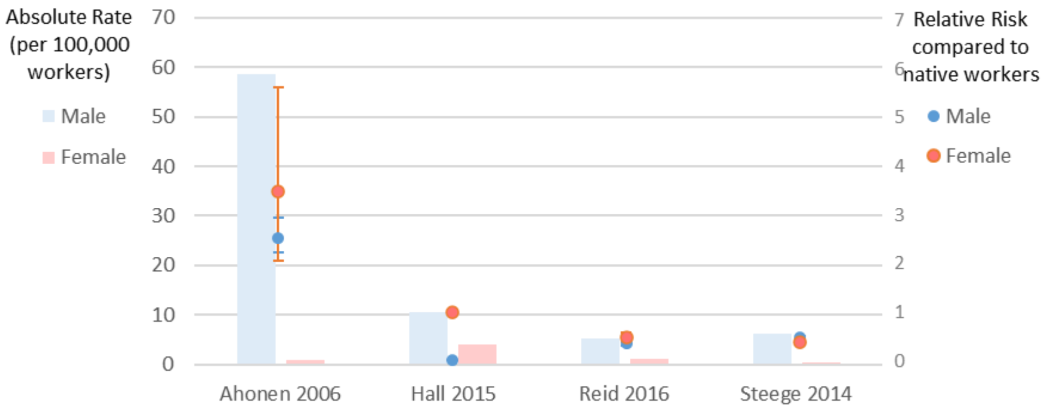

*error bars are 95% confidence intervals, where available

S8. Sensitivity analyses

A. Excluding low quality studies in the meta-analysis of fatal occupational injury

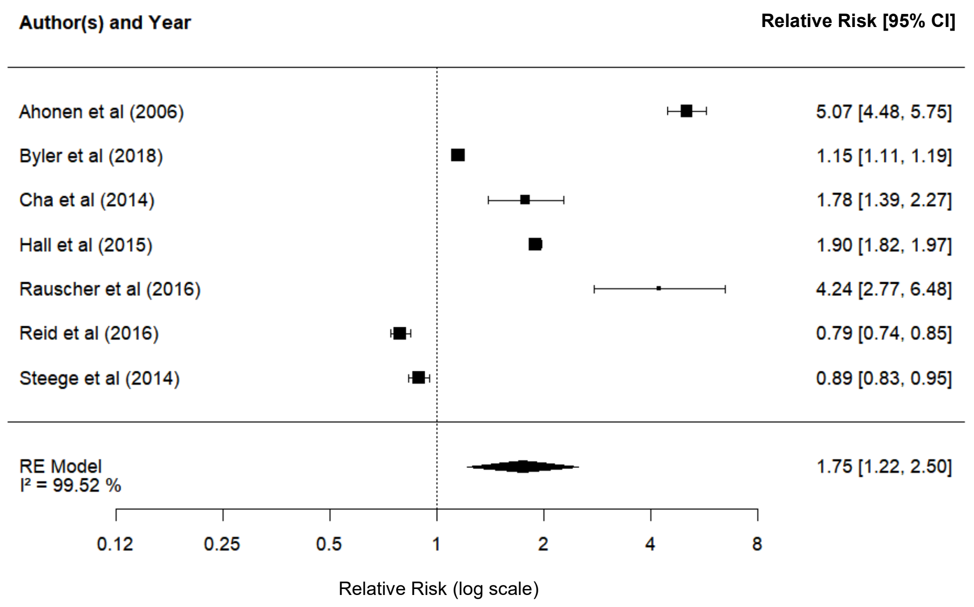

B. Using fixed effects model in the meta-analysis for fatal occupational injury

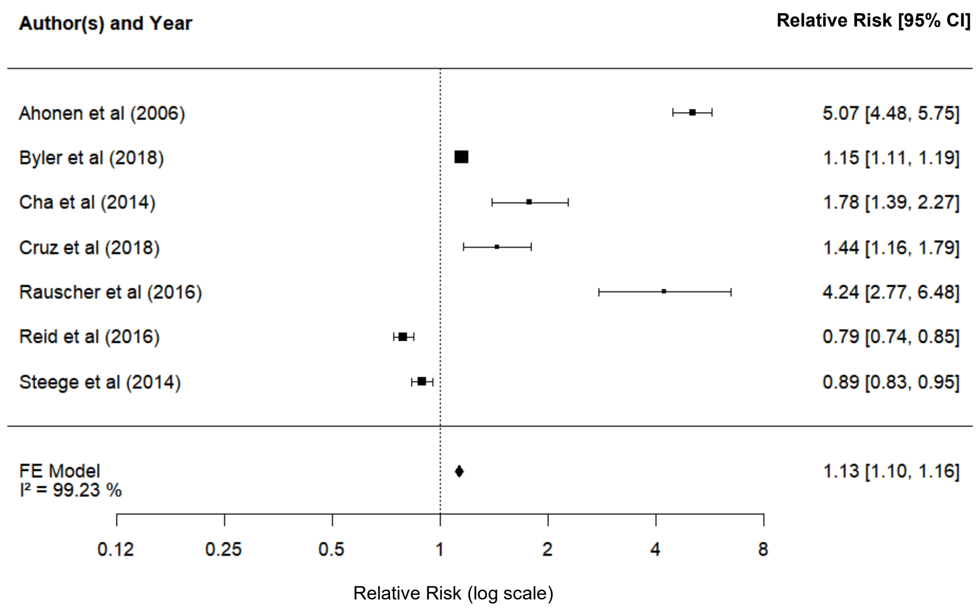

C. Excluding medium and low quality studies in the meta-analysis of fatal occupational injury

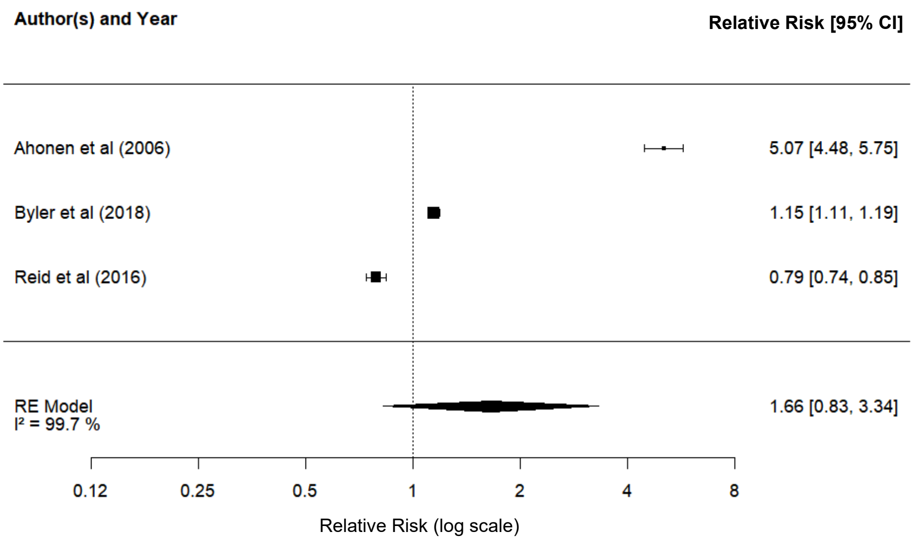

D. Using fixed effects model in the meta-analysis for all-cause mortality

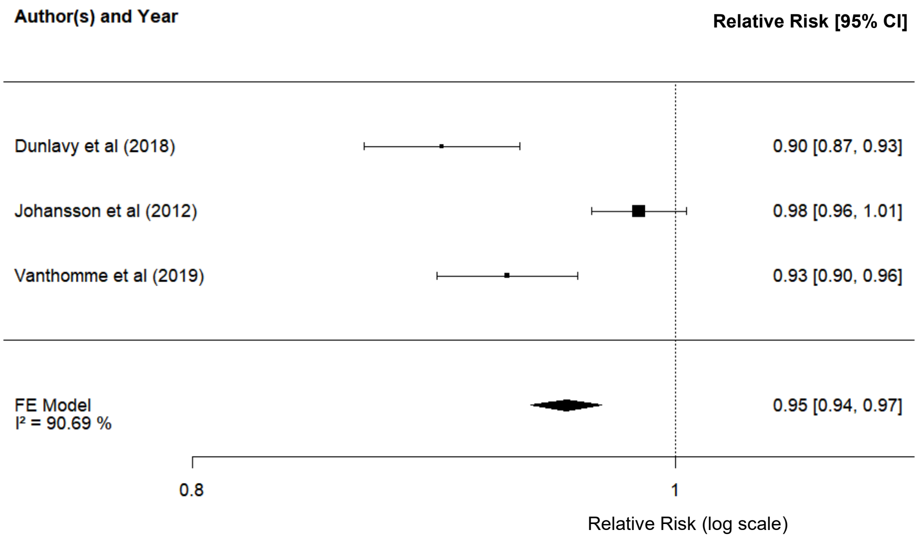

S9. Funnel plots

A. Funnel plot of RR for fatal occupational injury

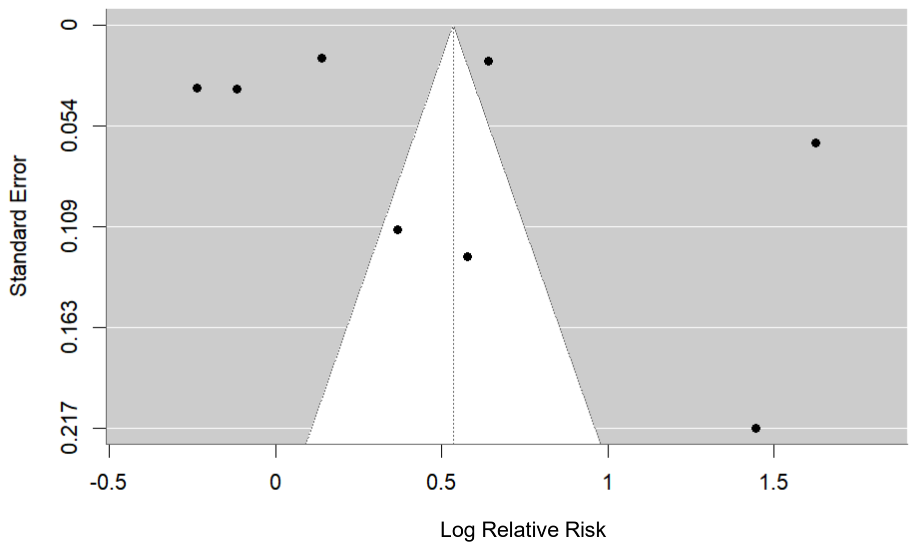

B. Funnel plot of RR for all-cause mortality

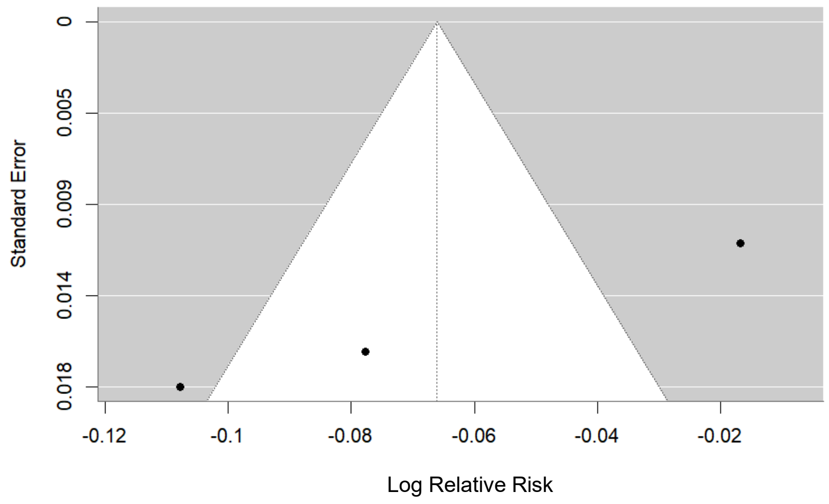

S10. PRISMA 2020 checklist

Section and Topic	Item #	Checklist item	Location where item is reported
**TITLE**			
Title	1	Identify the report as a systematic review.	Title
**ABSTRACT**			
Abstract	2	See the PRISMA 2020 for Abstracts checklist.	Abstract
**INTRODUCTION**			
Rationale	3	Describe the rationale for the review in the context of existing knowledge.	Introduction para 1-2
Objectives	4	Provide an explicit statement of the objective(s) or question(s) the review addresses.	Introduction para 2
**METHODS**			
Eligibility criteria	5	Specify the inclusion and exclusion criteria for the review and how studies were grouped for the syntheses.	Methods under "Search strategy and selection criteria" and "Inclusion/exclusion criteria and definitions"
Information sources	6	Specify all databases, registers, websites, organisations, reference lists and other sources searched or consulted to identify studies. Specify the date when each source was last searched or consulted.	Methods under "Search strategy and selection criteria"
Search strategy	7	Present the full search strategies for all databases, registers and websites, including any filters and limits used.	Methods under "Search strategy and selection criteria" and Supplementary Material S1
Selection process	8	Specify the methods used to decide whether a study met the inclusion criteria of the review, including how many reviewers screened each record and each report retrieved, whether they worked independently, and if applicable, details of automation tools used in the process.	Methods under "Search strategy and selection criteria"
Data collection process	9	Specify the methods used to collect data from reports, including how many reviewers collected data from each report, whether they worked independently, any processes for obtaining or confirming data from study investigators, and if applicable, details of automation tools used in the process.	Methods under "Data analysis"
Data items	10a	List and define all outcomes for which data were sought. Specify whether all results that were compatible with each outcome	Methods under

Section and Topic	Item #	Checklist item	Location where item is reported
		domain in each study were sought (e.g. for all measures, time points, analyses), and if not, the methods used to decide which results to collect.	"Inclusion/exclusion criteria and definitions" and Table 1
	10b	List and define all other variables for which data were sought (e.g. participant and intervention characteristics, funding sources). Describe any assumptions made about any missing or unclear information.	Methods under "Data analysis"
Study risk of bias assessment	11	Specify the methods used to assess risk of bias in the included studies, including details of the tool(s) used, how many reviewers assessed each study and whether they worked independently, and if applicable, details of automation tools used in the process.	Methods under "Quality assessment"
Effect measures	12	Specify for each outcome the effect measure(s) (e.g. risk ratio, mean difference) used in the synthesis or presentation of results.	Methods under "Data analysis"
Synthesis methods	13a	Describe the processes used to decide which studies were eligible for each synthesis (e.g. tabulating the study intervention characteristics and comparing against the planned groups for each synthesis (item #5)).	Methods under "Data analysis"
	13b	Describe any methods required to prepare the data for presentation or synthesis, such as handling of missing summary statistics, or data conversions.	Methods under "Data analysis"
	13c	Describe any methods used to tabulate or visually display results of individual studies and syntheses.	Methods under "Data analysis"
	13d	Describe any methods used to synthesize results and provide a rationale for the choice(s). If meta-analysis was performed, describe the model(s), method(s) to identify the presence and extent of statistical heterogeneity, and software package(s) used.	Methods under "Data analysis"
	13e	Describe any methods used to explore possible causes of heterogeneity among study results (e.g. subgroup analysis, meta-regression).	Methods under "Data analysis"
	13f	Describe any sensitivity analyses conducted to assess robustness of the synthesized results.	Methods under "Data analysis"
Reporting bias assessment	14	Describe any methods used to assess risk of bias due to missing results in a synthesis (arising from reporting biases).	Supplementary material S4
Certainty assessment	15	Describe any methods used to assess certainty (or confidence) in the body of evidence for an outcome.	NA
**RESULTS**			
Study selection	16a	Describe the results of the search and selection process, from the number of records identified in the search to the number of studies included in the review, ideally using a flow diagram.	Results under "Overview of included studies" and Figure 1
	16b	Cite studies that might appear to meet the inclusion criteria, but which were excluded, and explain why they were excluded.	Figure 1

Section and Topic	Item #	Checklist item	Location where item is reported
Study characteristics	17	Cite each included study and present its characteristics.	Table 1
Risk of bias in studies	18	Present assessments of risk of bias for each included study.	Supplementary material S2
Results of individual studies	19	For all outcomes, present, for each study: (a) summary statistics for each group (where appropriate) and (b) an effect estimate and its precision (e.g. confidence/credible interval), ideally using structured tables or plots.	Results sub-sections, Figures 2-5
Results of syntheses	20a	For each synthesis, briefly summarise the characteristics and risk of bias among contributing studies.	Results under "Overview of included studies" and sub-sections
	20b	Present results of all statistical syntheses conducted. If meta-analysis was done, present for each the summary estimate and its precision (e.g. confidence/credible interval) and measures of statistical heterogeneity. If comparing groups, describe the direction of the effect.	Results sub-sections and Figures 2-5
	20c	Present results of all investigations of possible causes of heterogeneity among study results.	Results sub-sections Figures 2-5
	20d	Present results of all sensitivity analyses conducted to assess the robustness of the synthesized results.	Supplementary material S3
Reporting biases	21	Present assessments of risk of bias due to missing results (arising from reporting biases) for each synthesis assessed.	Results not presented
Certainty of evidence	22	Present assessments of certainty (or confidence) in the body of evidence for each outcome assessed.	NA
**DISCUSSION**			
Discussion	23a	Provide a general interpretation of the results in the context of other evidence.	Discussion para 1-3
	23b	Discuss any limitations of the evidence included in the review.	Discussion para 4
	23c	Discuss any limitations of the review processes used.	Discussion para 4
	23d	Discuss implications of the results for practice, policy, and future research.	Discussion para 5
**OTHER INFORMATION**			
Registration and protocol	24a	Provide registration information for the review, including register name and registration number, or state that the review was not registered.	Methods under "Search strategy and selection criteria"

Section and Topic	Item #	Checklist item	Location where item is reported
	24b	Indicate where the review protocol can be accessed, or state that a protocol was not prepared.	Methods under “Search strategy and selection criteria”
	24c	Describe and explain any amendments to information provided at registration or in the protocol.	NA
Support	25	Describe sources of financial or non-financial support for the review, and the role of the funders or sponsors in the review.	Methods under “Role of funding source”
Competing interests	26	Declare any competing interests of review authors.	Declaration of interests
Availability of data, code and other materials	27	Report which of the following are publicly available and where they can be found: template data collection forms; data extracted from included studies; data used for all analyses; analytic code; any other materials used in the review.	Data sharing
